# Information Extraction and Population Estimates of Settlements from Historic Corona Satellite Imagery in the 1960s

**DOI:** 10.3390/s21072423

**Published:** 2021-04-01

**Authors:** Dimitris Stratoulias, George Grekousis

**Affiliations:** 1 Informetrics Research Group, Ton Duc Thang University, Ho Chi Minh City, Vietnam; dimitris.stratoulias@tdtu.edu.vn; 2 Faculty of Applied Sciences, Ton Duc Thang University, Ho Chi Minh City, Vietnam; 3 Department of Urban and Regional Planning, School of Geography and Planning, Sun Yat-sen University, Guangzhou 510275, China; 4 Guangdong Key Laboratory for Urbanization and Geo-simulation, Sun Yat-sen University, Guangzhou 510275, China

**Keywords:** Corona mission, remote sensing, historical GIS, feature extraction, segmentation, texture, settlements, human population, agriculture, field delineation

## Abstract

The Corona satellite program was a historic reconnaissance mission which provided high spatial resolution panchromatic images during the Cold War era. Nevertheless, and despite the historic uniqueness and importance of the dataset, efforts to extract tangible information from this dataset have primarily focused on visual interpretation. More sophisticated approaches have been either hampered or unrealized, often justified by the primitive quality of this early satellite product. In the current study we attempt to showcase the usability of Corona imagery outside the context of visual interpretation. Using a 1968 Corona image acquired over the city municipality of Plovdiv, Bulgaria, we reconstruct a panchromatic 1.8 m spatial resolution georegistered image with a relative displacement Root Mean Square Error (RMSE) of 6.616 (for x dimension) and 1.886 (for y dimension) and employ segmentation and texture analysis to discern agricultural parcels and settlements’ footprints. Population statistics of this past era are retrieved from national census and related to settlements’ footprints. An exponential relationship between the two variables was identified by applying a semi-log regression. The high adjusted R^2^ value found (76.54%) indicates that Corona images offer a unique opportunity for population data analysis of the past. Overall, we showcase that the Corona images’ usability extends beyond the visual interpretation, and features of interest extracted through image analysis can be subsequently used for further geographical and historical research.

## 1. Introduction

Corona, Argon, and Lanyard have been the first constellation of imaging reconnaissance satellites which acquired over 860,000 images over eastern Europe and Asia between 1960 and 1972 [1]. In 1995, the image archive was declassified [2] giving rise to one of the largest declassification projects in American history [3]. The availability of satellite imagery during the Cold War era has been an important addition in the information base of observing phenomena and changes of the mid last century. The importance of the Corona dataset is further underpinned by the 12 year long time span of the missions and the fine spatial resolution (1.8 m). Consequently, an interest was initiated by scientists and the public in exploring the usability of Corona imagery, primarily based on visual interpretation. Nevertheless, only a few studies have attempted to induce remote sensing and photogrammetric algorithms in the analytical context. This fact might be partially justified by the primitive nature of the Corona system, the consequent large image distortions and inaccuracies, and the lack of coexisting ground truth datasets.

The most common use of the Corona images to date has been the visual interpretation and digitization (e.g., [4,5,6,7,8,9,10,11,12]). This analytical context has been used in applications focusing primarily on land use and land cover, terrain analysis, and archaeology [13]. In regard to land use and land cover mapping, Tappan et al. [14] used Corona and Landsat imagery to map the changes that occurred in west-central Senegal between the early 1960s and the mid-1990s. In a similar effort, Tappan and McGahuey [15] analyzed land use and land cover trends in relation to environmental and agricultural conditions from 1965 until 2001 in southern Mali. Saleem et al. [16] classified synergistically Corona and Landsat images successfully with the maximum likelihood classifier in the context of mapping long-term land use and land cover changes. Hepcan [10] studied landscape pattern changes and connectivity in Urla district, Turkey based on data from the Corona (year 1963) and the Advanced Spaceborne Thermal Emission and Reflection Radiometer (ASTER) (year 2005) satellites. Sohn et al. [11] showcased the monitoring of urban expansion and water-bodies in Seoul, South Korea by integrating Corona Keyhole-4B (KH-4B) images while Andersen [12] mapped individual trees in the Eastern Desert of Egypt based on Corona KH-4A images. Brinkmann et al. [17] looked into landscape transformation in West Africa over the last 50 years.

Ruelland et al. [18] mapped land cover dynamics in Mali with Corona, Landsat Thematic Mapper (TM) and Satellite Pour l’Observation de la Terre 5 (SPOT 5) images, while Ruelland et al. [19] extended the aforementioned dataset by adding aerial photos and Landsat Multispectral Scanner (MSS) in Sahel from 1952–2003 for comparing three classification methods, namely pixel-based, object-based and in-screen digitizing; their findings suggest visual interpretation provided the most valid and coherent results in this multi-source approach. Similar observations to the latter have been reported by several studies which reckon that visual digitization provides superior results in comparison to digital image classification or that digitization has been felt as a better approach than image classification [14] or that on-screen interpretation was a necessity [17]. For instance, Hepcan [10] in a multi-temporal study involving Corona and ASTER data, he proceeded with digitizing the Corona image and carrying out supervised classification on the latter. The reason behind this suggestion is the limited spectral capabilities of the Corona sensor; however, a few late studies have demonstrated efficient use of other image manipulation techniques, with a notable study by Saleem et al. [16] who successfully used textural analysis as an input to a classification scheme.

Corona images have been largely used in archaeology [20]. For instance, Ur [9] used Corona imagery acquired over northern Mesopotamia to identify ancient road networks; he suggests that, in the absence of historical aerial photography, Corona is a useful tool for the identification of landscape features. For a more generic recent overview of the developments in the usability of geospatial technology in archaeology the reader is directed to Comer and Harrower [21] and Tapete [22].

Another notable application, underpinned by the fact that systems KH-4 (A and B) carried forward and backward looking cameras [13], is this of a Digital Elevation Model (DEM) generation. For instance, Schmidt et al. [23] used such stereoscopic image pairs to derive a DEM over a mountainous area in Morocco and reported 9 m planimetric and 20 m vertical accuracies. Focusing on the same region, Altmaier and Kany [24] generated a DEM with 3 m planimetric and 10 m vertical accuracies. Sohn et al. [25] present a 10 m grid interval DEM with horizontal and vertical ground accuracy of ± 4 m. Casana and Cothren [26] generated a DEM with the same grid interval over the Euphrates valley, Syria by treating sub-images of the scanned film (i.e., no larger than 35 × 35mm) as frame camera images. Other studies have been published where a DEM is generated from Corona images, such as the ones from Gheyle et al. [27] and Goossens et al. [28] both in Altai Mountains, Siberia, and Galiatsatos et al. [29] in Orontes valley, Syria. Sohn et al. [11] obtained 1.5 pixel level of horizontal accuracy and 2 pixels level of vertical accuracy when using Corona KH-4B imagery.

Various other applications have been documented. For example, Goerlich et al. [30] estimated glacier mass changes between 1964 and 1980 in Ak-Shirak, Kyrgyzstan. In another geological study, Deroin and Buslov [31] studies the 2003 Altai earthquake-triggered geomorphological changes in Gorny Altai, Siberia using a variety of Earth Observation data including Corona images. Agapiou et al. [32] produced a pan-sharpened product over Cyprus based on a Corona and a Landsat-8 image with the aim to improve the interpretation of the greyscale image. Last, Lorenz [33] used Corona KH-4A panchromatic and Landsat TM satellite imagery to study the geology in the Russian high Arctic.

Despite the demonstrated usefulness and consequent opportunities that Corona imagery opens, there are important limitations that need to be overcome to extract information, qualitative or quantitative, out of these types of images. The panoramic camera on board Corona inherits several types of distortions, which augment towards the end of each photograph and are not encountered in typical frame cameras. It is perhaps these technical challenges that have discouraged widespread sophisticated use of this unprecedented historic archive and use it instead simply as a reference for visual interpretation. Moreover, quality inconsistency across images and mainly across missions, appeals for advanced pre-processing techniques for the inaccuracies and uncertainties such as fuzzy methods for gray-level image contrast enhancement (e.g., [34,35]).

Primarily, the lack of basic acquisition information and metadata is hindering the use of mainstream satellite image processing. For instance, the time of acquisition is essential in earth observation as it determines the solar position at the moment of image acquisition, which consequently, has an effect on the appearance of objects in satellite imagery. The latter is especially prominent in very high spatial resolution (VHSR) satellite imagery as demonstrated by De Laet et al. [36] when comparing illumination effects for two different VHSR sources in the context of identifying archaeological features. On this note, Fowler [37] has demonstrated a model to determine the acquisition time specifically for Corona satellite images. As a second example, in classification exercises, several variables can be input in an image classification problem such as spectral signatures, vegetation indices, transformed images, ancillary data, multitemporal or multisensor images, and textural or contextual information [38]. From all the above, it is apparent that, for the case of a single panchromatic image, any manipulation of multi-spectral information, as is the case for the first three variables, is not applicable. Moreover, remotely sensed imagery during the same period of time from the same or a similar sensor might be difficult to acquire, if non-existent. Furthermore, reliable and accessible ancillary data, dating back to the 1960s and 1970s, such as the ones from Hepcan [10] who used aerial photographs from 1957 to carry out the accuracy assessment of a 1963 Corona image, may be difficult to acquire. As a matter of fact, several studies are using contemporary field data for validation purposes of Corona image processing results, such as the study conducted by Andersen [12] who used field data from 2003 to verify the existence of trees mapped from a Corona KH-4A image acquired in 1965.

It therefore becomes apparent that, for remotely sensed images of this era and specifications, textural information may have practical image post-processing applications. Texture has been shown to be useful in analyzing several VHSR data; for instance, Alonso et al. [39] presented a case study in Spain based on VHSR Ikonos satellite data, where inclusion of textural information from the panchromatic band improved the classification accuracy by 3.4%. However, and despite the fact that the combination of VHSR and panchromatic nature of Corona underpins the extraction of texture as a consequent product, this is a field that has been largely unexplored. Only lately efforts started to spring up, with notable the application from Saleem et al. [16] who used textural analysis as an input in a classification scheme.

A breadth of applications are found in the literature review. Nevertheless, there have not been many studies linking information extracted from Corona images with other data, such as demographics, reported during the mid-20th century. However, the significance of extracting population estimates from satellite images has been well documented, especially for historical census data that are often limited in their precision [40]. Moreover, the usability of the Corona images in the context of remote sensing analysis has been primitive and undervalued. The current study contributes to the existing literature and attempts to fill the above-mentioned gaps in two ways. First, it evaluates the feature extraction capability from Corona imagery based on segmentation and texture analysis. We showcase that more sophisticated techniques, other than visual interpretation, are applicable to primitive Corona imagery. Second, features of settlement areas, extracted from Corona, are coupled to census data to investigate whether Corona images can be used for estimating population. An application of regression analysis demonstrates that the settlement area extracted from Corona image is a good predictor of population estimates. Overall, we claim that, following careful and tailored processing steps of Corona imagery, feature extraction, and linkage to other concurrent available information, is feasible and this first Earth Observation satellite largely unexplored dataset can be a basis of historic information during the Cold War era.

## 2. Materials and Methods

### 2.1. Study Area

**Figure 1:**
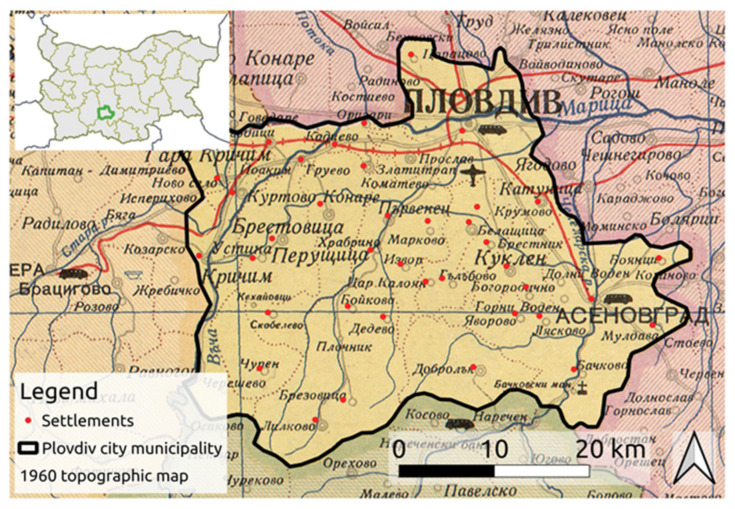
Study area of the city municipality of Plovdiv (black outline) overlaid on a georeferenced historic administrative map from 1960 and the location of the sampled 39 settlements (red points). Inset image presents the relative location of the city municipality of Plovdiv in the context of Bulgaria. The historic background map was issued by the Bulgarian State Directorate of Geodesy and Cartography under the title of “Administrative map of People’s Republic of Bulgaria”, ID KpIV314, designed in 1960 and printed in 1963.

Between 1959 and 1987, Bulgaria defined three administrative units with the special statute of “city municipality” (gradska obshtina), namely those of Sofia (the capital), Varna, and Plovdiv. “City municipalities” were directly administered by the central government, whereas the provinces (okrugs) were administered by local governmental agencies (Decree № 29 of 22 January 1959 (promulgated in Gazette officielle, Issue 7, 23 January 1959)). The current study focuses on the municipality administrative unit encompassing 45 smaller settlements including the Plovdiv city (Figure 1). 

Bulgaria’s demography has been characterized by steep population decline at a national scale since 1989, while the phenomenon at rural areas started at 1975, and at regional level in 1930, as a consequence of the constant flow of the rural population to the cities [41]. The phenomenon has been intense; for instance, between 1992 and 2001 the rural population decreased 11.1% [42], a process which consequently depopulated large geographic territories, and especially mountainous regions [43]. The reasons of the undergoing rapid demographic changes are notably the low fertility rate [44] and high death rate, especially in the beginning of the 21st century [45], and consequently a negative natural increase rate, distinct population aging, and emigration of young people of fertile and work-productive age [46]. The future outlook is discouraging as it was recently estimated by the UN Population Division that by 2050 Bulgaria will shrink by 23% in total population, which is the largest decline worldwide [47]. Under this structure, little has changed in regard to settlement sprawl and agricultural activity within the country since the 19th century [48].

### 2.2. Corona Satellite Imagery

**Figure 2:**
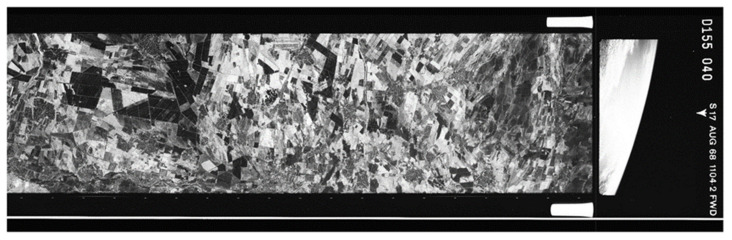
Tile of a Corona product as delivered by the United States Geological Survey (USGS) in 4 complementary parts. A scanner is used to scan the archived monochromatic camera film which contains the Corona image.

Corona satellite imagery was employed to reconstruct the landscape of the study area in the year 1968. Corona images over Plovdiv are available from 18 August 1960 until 24 July 1970, with a large discrepancy in radiometric and spatial resolution between missions; the latest missions, at the end of the 1960s, inheriting the best image quality. In the current study, 4 deliveries from mission 1104-2, acquired at 150 km altitude, on 17 August 1968 were used to cover the entire study area with a total cloud coverage of approximately 5%. The KH-4B system employed in the specific mission carried two panoramic film cameras with a 609.602 mm focal length (Figure 2) and a separation angle of 30° between the first (forward) and the second (aft) camera, and delivered images of a single panchromatic band (500–900 nm) at 2.75 m spatial resolution (Table 1). It contained minimal metadata or ephemeris information, low signal-to-noise ratio and important radiometric and spatial distortions, relative to the contemporary satellite systems; nevertheless, it delivered the best imagery to date on any KH-4 systems [49]. Each digital image is in essence scans of the source film and a single delivery contains 4 adjacent scans of the film in TIFF format. The data were retrieved from the Earth Resources Observation and Science (EROS) Center of the United States Geological Survey (USGS).

### 2.3. Census

Decennial census data inventorying Bulgarian population statistics per settlement are available for the years 1965, 1975, 1985, 1992, 2001, and 2011 while the next census is scheduled for 2021. Agricultural aggregated data are available for the year 1968. Each settlement is attributed a unique Unified Classification of Administrative Territorial and Territorial Units (UCATTU, or otherwise known as EKATTE) number and the exact geolocation is known based on data available from the Bulgarian National Statistical Institute (National Register of Populated Places 2020).

### 2.4. Methodology

**Figure 3:**
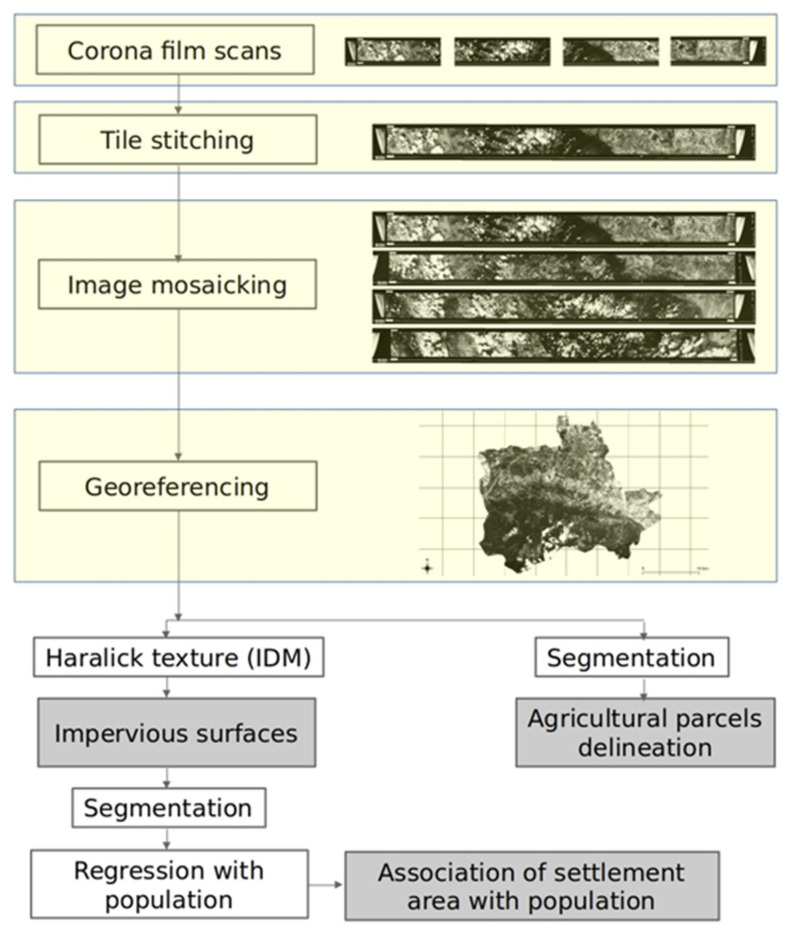
Workflow of the Corona satellite image processing and population analysis. Yellow fields correspond to satellite image mainstream pre-processing, transparent fields to image manipulation and grayed fields in workflow products presented in the results section.

The methodology followed in the current study is depicted in Figure 3. A historic topographic map from the year 1960 (KpIV314) was used to extract the administrative boundaries of this era (Figure 1). The map was georeferenced based on 21 Ground Control Points (GCP) selected on top of the approximate location of major cities of the administrative divisions. Thereafter, a polygon encompassing the Plovdiv city-municipality was delineated manually and defined the study area.

The Corona scans were processed to reconstruct a satellite image depicting the land cover of the year 1968. The 4 tiles from each of the Corona deliveries were first stitched together using the Adobe Photoshop 2020. The 4 Corona composites were then georeferenced with 30 Ground Control Points (GCP) selected from web map services; GCPs at the upper and lower part of the image were primarily selected in order to maintain coherence between the individual tiles. Road intersections within the settlements were used as tie points to adhere to high georeferencing accuracy within and around the settlements, as well as because these are GCPs that are easily discernible and have not changed in the last 60 years. The Thin Plate Spline (TPS) transformation and the Nearest Neighbor (NN) resampling method were used, as the comparison between the two indicated that the TPS provides more accurate results.

It is important to note that, due to intense geometric distortions, a few studies have deliberately not attempted to annotate geographical coordinates (e.g., [50]) in the light of preserving the raw information. The current study, however, is based on the comparison and synergistic analysis with other spatial products and hence geographical referencing is necessary.

We then projected the image onto the World Geodetic System (WGS) 84 geographic coordinate system. Thereafter, the void parts of the image were eliminated by attributing nodata values to the very low values (corresponding primarily to the black parts of the film) and using the latter result as a guide to select a bounding box encompassing the majority of the image. Each georeferenced tile was then subset with the corresponding bounding box. Then the 4 processed product tiles were merged and finally clipped with the study area vector to derive the composite per-processed image. Finally the image was projected to the Universal Transverse Mercator (UTM) 34N cartographic coordinate system so that metric surface area estimation from the imagery can be realized.

Thereafter, a segmentation algorithm was applied to investigate whether agricultural parcels can be delineated based on object based image analysis. A total of four segmentation algorithms were investigated, namely the mean-shift (spatial radius (5–50), range radius (5–30), and minimum region size (100–2500)), connected components (distance (5–20) and minimum object size (90–200)), watershed (depth threshold (0.005–1) and flood level (0.005–2)), and morphological profiles based segmentation (profile size (5–200)). The parameters for each value were selected incrementally and after visual evaluation of each result.

The Haralick texture analysis was then applied as in similar studies (e.g., [39]). The features of energy, entropy, correlation, Inverse Difference Moment (IDM), inertia, cluster shade, cluster prominence, and Haralick correlation were extracted. The IDM was selected as the layer with the best discriminatory power after visual evaluation. A segmentation of the latter based on the connected components algorithm provided the segments of the impervious surfaces representing the settlements’ areas. In the case of five settlements, the segmentation did not distinguish sufficiently between the urban extent and the surrounding possibly agriculture activity, and therefore, we refined the segments manually. Finally, the settlements encompassed by the study area were extracted from the map product through a clipping operation and were judged against the census data through the EKATTE number.

In the last step of the methodology, the population data from the year 1965 were used as settlement proxy since this available census year is the closest to the Corona image acquisition date. It is hypothesized that the settlement population is directly related to settlement size. Scaling laws and the associated power models have been widely used to model the relationship between urban settlement area and population [51]; however, scaling relationships may not apply to small settlements like villages [52]. This is because settlements with higher densities lead to increasingly structured land use and the segregation of buildings, roads and other infrastructure [52,53]. For this reason, apart from the power model, we tested the exponential model as well. Results showed that the exponential model yielded a better fit than the power model. For this reason, we only present here the exponential model equation (Equation (1); [54]).

*P* = *ae^rs^* (1)

where *P* = represents the population, *s* the settlement area, *r* the growth rate, *a* is a constant.

Thus, the natural log of both sides of Equation (1) provides Equation (2):

*logP* = *loga* + *rs* (2)

A linear regression was used to model the relationship between *logP* and size *s* by estimating the *r* (slope) and the intercept *loga*. The slope reveals how much *logP* changes for a one-unit change in size.

The algorithmic development was established primarily on the Geospatial Data Abstraction Library (GDAL) and QGIS [55] while the segmentation was implemented with the Orfeo ToolBox [56]. Regression analysis was conducted with Matlab R2016a and specifically the fit linear regression model (fitlm) function in the Statistics Toolbox [57].

## 3. Results

**Figure 4:**
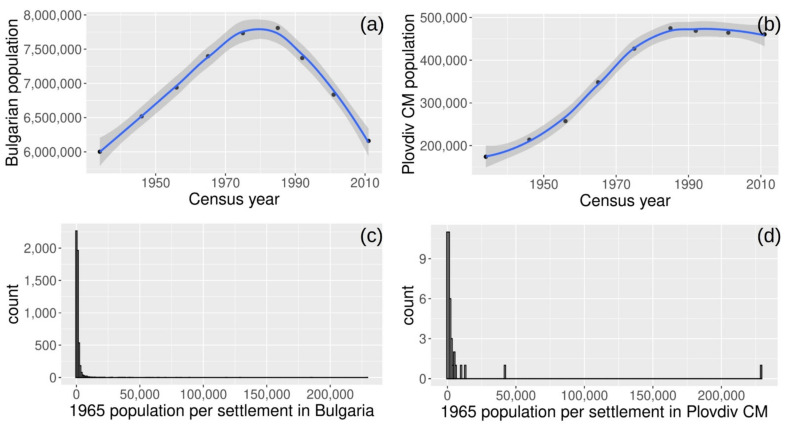
Temporal change in total population for Bulgaria (**a**) and per settlement within the Plovdiv City Municipality (CM) (**b**) based on Census decadal data and distribution of settlements (bin of 1000) for Bulgaria (**c**) and the study area (**d**) in the year 1965.

The census data were analyzed to provide an overview of the population trends in Bulgaria and the distribution of settlement sizes. The population progression over time for the whole country of Bulgaria and for the Plovdiv Municipality City are depicted in Figure 4. Moreover, the distribution of population per settlement reveals that the majority of the settlements contain a rather small population (<10,000 people) with the city of Plovdiv being disproportionately large and, therefore, excluded from further inclusion in the processing.

The results from the georeferencing exercise of the Corona image are presented in Table 2. The TPS transformation yielded the most accurate results with a residual error of 1–5 m which is a precision that satisfied the delineation of impervious surfaces, as per purpose of this study.

**Figure 5:**
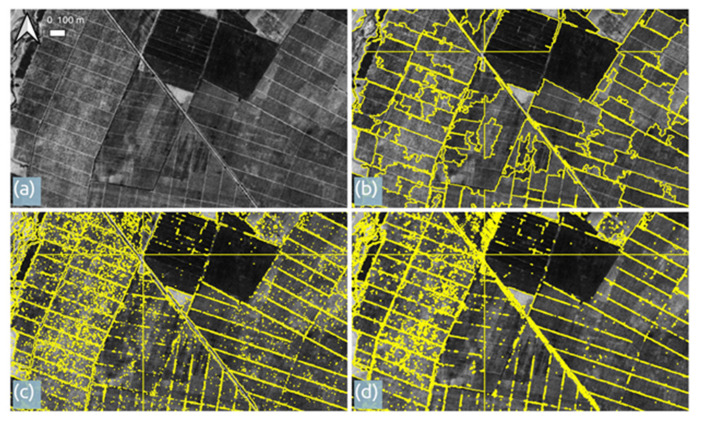
A typical agricultural area in Plovdiv as depicted in the Corona post-processed image (**a**) and best examples of segmentation corresponding to agricultural fields from the mean-shift (**b**), connected components (**c**) and watershed (**d**).

The image segmentation is presented in Figure 5. Results varied depending on the thresholds selected for each algorithm; the best examples are presented which correspond to the mean-shift (spatial radius (5), range radius (8) and minimum region size (1000)), connected components (distance (12) and minimum object size (90)), and watershed (depth threshold (0.1) and flood level (0.2)). Morphological profiles based segmentation did not yield satisfactory results and hence is not presented herewith.

**Figure 6:**
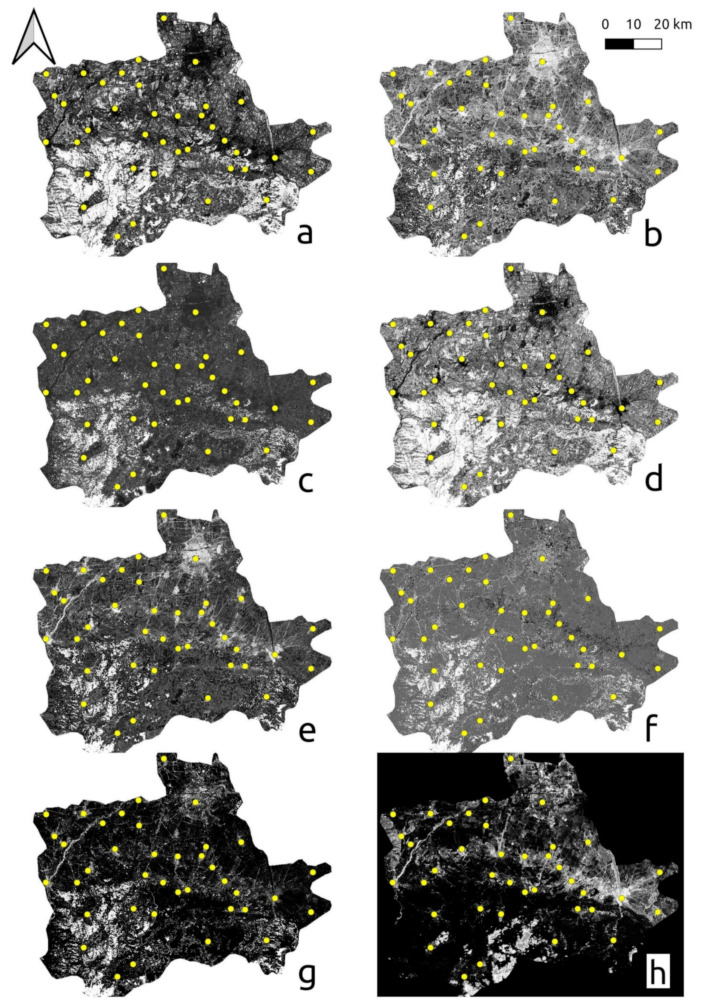
Haralick texture analysis of energy (**a**), entropy (**b**), correlation (**c**), Inverse Difference Moment (IDM) (**d**), inertia (**e**), cluster shade (**f**), cluster prominence (**g**), and Haralick correlation (**h**).

The results from the texture analysis are presented in Figure 6. The IDM was judged as the layer with the maximum contrast between impervious surfaces and the surrounding agriculture and natural environment. The layers of energy, entropy, and inertia are also inheriting high discriminatory power in regard to impervious surfaces. On the other hand, correlation, cluster shade, and cluster prominence did not perform satisfactorily.

Lastly, to assess the relationship between the Settlement area extracted from the textural analysis and Population for the settlements situated within the Plovdiv city municipality, we tested the power and the exponential models (Table 3).

**Figure 7:**
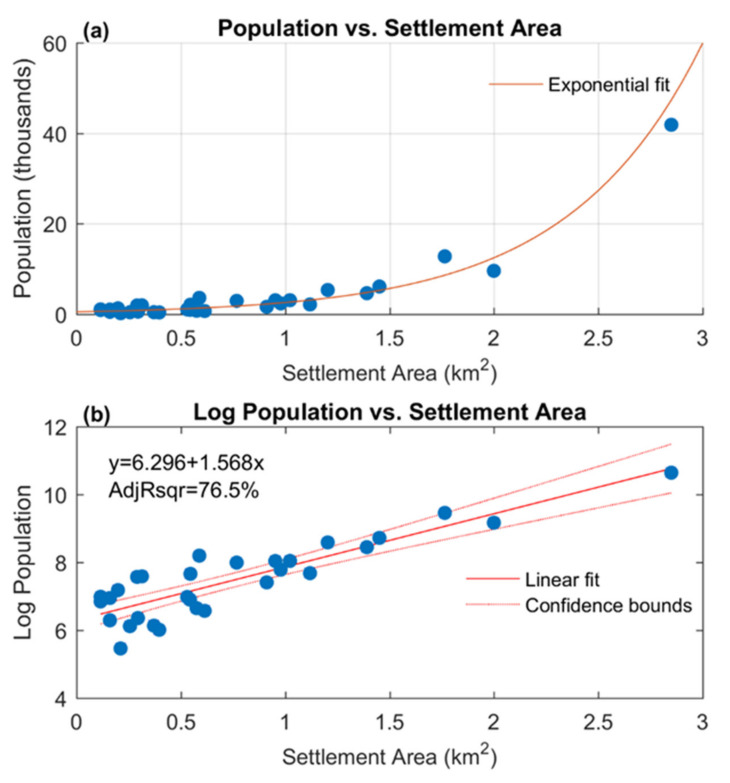
An exponential trend is identified between *Population* (as derived from Census data) and Settlement area (as derived from the Corona image) (**a**) and Linear regression of Log Population vs. Settlement area (**b**) reveals a statistically significant linear relationship with a good fit (adjusted R^2^ = 76.5, *p*-values < 0.001 for F-test (significance) and t-test (coefficients), confidence level of the bounds is 95%).

Results showed that the exponential model yields a better fit (adjusted R^2^ = 76.54%) than the power model (adjusted R^2^ = 59.62%). To estimate the exponential model parameters we fitted a simple linear regression model (Equation (2)) to the observed data with response logPopulation and predictor Settlement Area, which outputs an estimate for slope r = 1.568 (*p*-value = 1.5792 × 10^−10^) and intercept a = 6.296 (*p*-value = 3.1495 × 10^−26^), (Figure 7), and F-test = 95.6 (*p*-value = 1.58 × 10^−10^). A slope or r = 1.568 indicates that an increase in 1 km^2^ results in population growth by 100e^r^% = 479.7%. From the demographic perspective, this means that a settlement 1 km^2^ larger than another has nearly five times larger population. As the linear fit of the model is high (adjusted R^2^ = 76.54%), we argue that it is feasible to estimate the population of similarly-sized settlements in nearby municipalities and provinces using Corona images of the same period.

## 4. Discussion

Figure 4 presents the progression of population for the whole country and a bell-shaped distribution is observed. An increase from the year 1940 until 1980 is evident and thereafter a decrease until 2010, which continues to take place to date. When isolating the Plovdiv city municipality the same trend is observed until 1980, however, thereafter the population remains stable perhaps attributed to the urbanization of the major city of Plovdiv. Most importantly, the bar chart of Figure 4 presents the distribution of settlements in Bulgaria and Plovdiv city municipality. It becomes apparent that in both cases, the majority of the settlements are of the size of less than 10,000.

**Figure 8:**
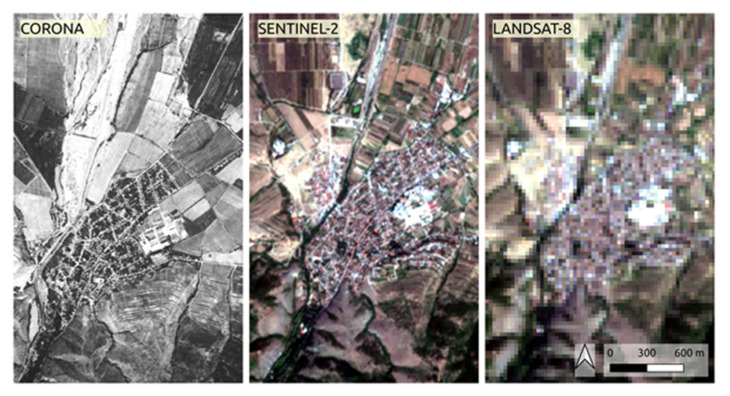
Comparison of the spatial resolutions of a 2.6m panchromatic Corona image acquired on 17 August 1968 (scene ID DS1104-2155DF040 available from the Earth Resources Observation and Science (EROS) website), a 10 m true-color representation Sentinel-2B image acquired on 29 August 2019 (scene ID S2B_MSIL1C_20190829T090559_N0208_R050_T35TLG_20190829T112604 available from the Copernicus Open Access Hub) and a 30 m true-color representation Landsat-8 image acquired on 21 August 2019 (scene ID LC81830312019233LGN00 freely available from the EarthExplorer website) over the settlement of Parvenets, Bulgaria. All image data sources presented are freely available to download from the respective sources.

Corona satellite images are primitive compared to modern satellite systems in regard to radiometric resolution, distortions and accuracy. Moreover, only a panchromatic band is available and consequently the quantification of land cover information (such as vegetation traits) through spectral indices or multi-band classification schemes cannot materialize. Nevertheless, they have the unique vantage point of making available a land cover snapshot in a time of more than 50 years ago at a high spatial resolution. Figure 8 showcases depictions of Corona, Sentinel-2, and Landsat-8 satellite images (see also [58]) of a small settlement (i.e., Parvenets) with a population of 3105 inhabitants in the year 1965 (3652 in 2011). Spatial resolution differences are the most important constraint when comparing images of different satellite sensors [19]. In our case, it is apparent that certain details such as delineated roads, buildings, and agricultural parcels are inherited in the Corona imagery while they are less prominent in a contemporary Sentinel-2 image (spatial resolution 10m) and even diminish at the image delivered by Landsat-8 (30 m spatial resolution). This observation of the high spatial resolution and the consequent discernible textural characteristics is the anchor point of this current study which present the opportunities to use Corona imagery outside the scope of image interpretation.

A visual evaluation of the accuracy on the overlap between the two images indicates a good agreement in the locations around the GCPs, which were mostly selected within settlements of the study area. However, important displacements were observed in areas that are further away from the GCPs. A higher order polynomial is necessary in this case.

The Corona KH-4B inherits the finest spatial resolution of approximately 1.8 m at nadir in certain missions. Fine spatial resolution is especially important in the context of archaeology, however, De Laet et al. [36] in a study investigating extracting archaeological features from VHSR satellite images (i.e., Ikonos-2 and Quickbird-2) in Sagalassos, Turkey stresses the importance of both the spatial, as well as the spectral resolution. In the case of the Corona image, the latter is unfortunately a weak point both from the aspect of radiometry, as well as number of spectral channels. Nevertheless, Corona’s spectral domain covers the region 0.5–0.9 μm which is suitable for vegetation related spectral observations [59,60] while the fine spatial resolution is a prerequisite for inventorying smallholder agricultural fields [61,62] which is the agricultural practice established in Bulgaria. However, the single panchromatic band inherits a low Singal to Noise Ration (SNR) which is not adequate for spectral analysis; a comparison of the pixel values between major land cover classes (agriculture, settlements, natural vegetation) presented no discernible pattern. Moreover, unsupervised and supervised classification attempts undertaken did not yield important results (results not shown here). This might be perhaps attributed to the fine spatial resolution of the data source, as improved spatial resolution increases within-class variances, which results in high interclass spectral confusion as claimed by Gong et al. [63].

Classification accuracy is often the result of the concurrent high spectral and spatial resolution of the input data [64,65,66,67] which will be cumbersome to construct for the era that Corona imagery was available in the absent of concurrent multispectral data. Nevertheless, the spectral information deficit inherent in the Corona imagery might be circumvented by utilizing the fine spatial resolution which can provide the opportunity to extract features through segmentation and texture analysis.

The segmentation results indicate that, after fine tuning, agricultural parcels can be delineated based solely on Corona imagery. The mean-shift algorithm considers the texture of the crop within the field to derive border lines, which might be the effect of different soil humidity as expressed in the panchromatic band. The connected components and watershed algorithms seem to delineate the actual borderlines of the crop fields. In all 3 algorithms, defining a minimum region size had the advantage of identifying regions sufficiently small to discern smaller fields while at the same time large enough to avoid demarcation of the fields into smaller segments.

The exhibition of texture in VHSR satellite imagery has a potential for information extraction in land cover applications. Several spatial feature extraction methods have been developed, such as the gray level co-occurrence matrix (GLCM), the simple statistical transformation (SST) and the texture spectrum (TS). Integration of spatial features have shown to improve the classification accuracy of land use classification in comparison to when obtained by the use of the spectral images only [63]. VHSR panchromatic images are especially suitable for this purpose and GLCM has successfully used in this context (e.g., [68]). In our case, IDM, which is a measure of texture homogeneity, presented the results with the most discriminatory power in distinguishing impervious surfaces with the surrounding primarily vegetated environment. The fine-tuned segmentation over the IDM, revealed that segmentation can delineate urban structures; only in a few instances, the urban texture seems to be spectrally and texturally similar to the surrounding environment, and the segmentation resulted in an overestimation of the settlement area. While Corona allows the identification of generic land cover classes, Pacifici et al. [69] in a study using QuickBird and WorldView-1 sub-meter resolution images to classify the land-use based on textural metric, demonstrate the discrimination between different asphalt surfaces (namely roads, highways, and parking lots) from the textural information content.

The importance of extracting population estimates from satellite images has been widely acknowledged for incomplete and not regularly conducted censuses (i.e., in developing countries) and census data that are relatively old [70,71]. Population estimates through recently collected satellite data are vital to many aspects of public interventions ranging from public health to resources and services allocation [72,73]. On the other hand, historical data from censuses are often limited in their precision and geographical coverage. For this reason, a retrospective population estimate may offer a more accurate and complete assessment of the past [74]. Through historical satellite data, population can be estimated at more regular intervals than the decennial censuses, increasing temporal granularity. This allows for better monitoring population and built-up growth through time and more efficiently relating them to the same era’s historical and political context.

In the current paper, we show that due to the high quality in texture extraction (compared to other available historical satellite images), Corona images offer a unique opportunity for population data analysis in the 1960s and 1970s. In one of the very few similar studies conducted to date by Saleem et al. [16] with the aim to relate population increase from 1969 to 2014 and urban growth in the Kurdistan region, a positive exponential relationship was established (R^2^ = 0.89). Moreover, in a recent paper, Stratoulias and Kabadayı [75] have found an association (R^2^ = 0.54, *p* = 0.02) between census population statistics settlement area extracted from Corona and this can act as an indication of a positive association. In the current paper, we integrated a more sophisticated image processing workflow and a larger number of samples which resulted in a good fit (R^2^ = 0.76%), between population data referred to in the 1965 Bulgarian census for 30 settlements, and their settlement size as estimated in this research. This showcases that information extraction from Corona images is feasible, and more importantly that it can be associated with other historical socioeconomic data pertaining to the Cold War era.

## 5. Conclusions

Corona satellite images have long been available to the scientists and the public; nevertheless, the usability of this unique dataset and source of information for the Cold War era has been confined primarily in visually interpretation. The current paper presented the application of contemporary mainstream remote sensing image analytics, and mainly segmentation and texture extraction, for the purpose of linking qualitatively and quantitatively the information from Corona with land cover features. We showcase the usability of segmentation in delineating agricultural parcels and texture analysis in deriving impervious surfaces corresponding to settlements’ footprints over Plovdiv, Bulgaria. Efforts to correlate the latter with census population of that era indicated a strong association between the two variables (R^2^ = 0.76%), which we claim to be an argument for supporting the deployment of Corona imagery for urban land cover extraction and, subsequently, indirectly population estimates. The derivation of texture augments the information inherited within the Corona imagery. Moreover, Corona imagery, combined with more recent satellite imagery, allows for the creation of extended decadal time-scale series, as the one demonstrated by Tappan and McGahuey [15] who compiled a study from 1965 until 2001 in Southern Mali. The findings of the current study indicate research gaps that could become active fields of future research, such as the deployment of Corona for agricultural mapping (since the high spatial resolution is a per-requisite for smallholder agriculture which prevailed during this historic time), advanced segmentation, and classification schemes (e.g., [76,77]), application of fuzzy methods to compensate for imagery inconsistencies (e.g., [34,35]), and radiometric comparison with contemporary VHSR similar satellite systems (e.g., Quickbird, Ikonos, WorldView).

## Figures and Tables

**Table 1:** Technical specification of the Corona imagery used in the current study.

Attribute	Corona-Camera KH-4B
Image ID	DS1104-2155DF040, DS1104-2155DF041, DS1104-2155DF042, DS1104-2155DF043
Photogrammetric scanning resolution	7 Micron (3600 dpi)
Dynamic range	11 bit
Spatial resolution (georegistered)	2.75 m
Number of bands	1 (panchromatic)
Spectral bandwidth	500–900 nm

**Table 2:** Residuals and their relative displacement (mean, standard deviation and Root Mean Square Error (RMSE)).

Attribute	Value (m)
Mean_X_ of absolute residual values	5.288
Standard deviation_X_ of absolute residual values	4.026
Mean_Y_ of absolute residual values	1.875
Standard deviation_Y_ of absolute residual values	0.205
Min_X_ residual	−0.136
Max_X_ residual	17.1
Min_Y_ residual	1.416
Max_Y_ residual	2.318
RMSE_X_	6.616
RMSE_Y_	1.886

**Table 3:** Power and exponential models, relationship between the settlement area and population.

	Power Model	Exponential Model
R-squared	0.610	0.773
Adjusted R-Squared	0.596	0.765
Root Mean Square Error (RMSE)	0.725	0.552
F-statistic *p*-value	3.5 × 10^−7^	1.58 × 10^−10^

## Data Availability

The Corona satellite data presented in this study are openly available from the Earth Resources Observation and Science (EROS) Center of the United States Geological Survey (USGS).
